# Socio‐Structural Phenotypes and their Impact on Cognition in a Cohort of Hispanic/Latina/Latino Immigrants

**DOI:** 10.1002/alz70857_103530

**Published:** 2025-12-25

**Authors:** Lauren G Santos, Stella Garriga, Michael G Davenport, Jacob Fiala, Michael Marsiske, Franchesca Arias

**Affiliations:** ^1^ University of Florida, Gainesville, FL, USA

## Abstract

**Background:**

Hispanic/Latina(o) persons in the United States (U.S.) are at a unique intersection of cultural, social, and systemic experiences affecting health. While acculturation, the degree to which a person integrates aspects of their host country's culture, has been explored in cognitive outcomes, findings are inconclusive. Importantly, acculturation's effect on cognition is rarely explored in social and structural contexts. This study evaluated the association between acculturation and cognitive outcomes and examined whether social and structural factors affect this relationship.

**Method:**

A cross‐sectional analysis of Hispanic/Latina(o) participants (68% Female; *M_education_* = 10.01 ±  4.54; *M_age_* = 64 ± 7.99) enrolled in the Health and Aging Brain Study‐Health Disparities (HABS‐HD; *N* =  1,095) was conducted. Participants completed an acculturation measure, a social support scale, and a comprehensive neuropsychological workup. State‐level neighborhood disadvantage was calculated using the Area Deprivation Index (ADI). Analysis of variance (ANOVA) and multivariate ANOVA compared performance across seven cognitive domains based on acculturation level (low, moderate, high) and assessed whether cognitive performance differed by social support and State‐level ADI. K‐cluster analysis determined whether socio‐structural phenotypes captured cognitive outcomes.

**Result:**

Persons in the high acculturation tertile outperformed other groups in general cognition, processing speed, language, memory, and executive function (all *p* < .01). Social support was positively associated with general cognition, processing speed, language, memory, and executive function (all *p* < .01). ADI was inversely associated with general cognition, processing speed, memory, and executive function (all *p* < .01). An interaction between social support and ADI for language was found (*p* < .05). Three clusters were identified: (1) High Acculturation/Low Social Support/Moderate ADI, (2) Moderate Social Support/Lowest Acculturation/Highest ADI, and (3) High Social Support/Moderate Acculturation/Low ADI. Cluster 3 performed significantly better in general cognition (F(2, 725) = 15.43, p < .001, partial η^2^ = .025) and reading level (F(2, 725) = 143.14, p < .001, partial η^2^ = .19).

**Conclusion:**

The association between acculturation and cognition varied based on social and structural determinants of health. These findings underscore the importance of examining the contribution of these variables when assessing cognitive outcomes. Future studies are needed to determine whether interventions targeting social support and stressors in the built environment may promote cognitive health, particularly in the context of acculturation.

